# Decrement on Low-Frequency Repetitive Nerve Stimulation Is Not Synonymous With Myasthenia Gravis

**DOI:** 10.7759/cureus.114679

**Published:** 2026-08-17

**Authors:** Sidra Aurangzeb, Ali M Hassan, Sangeetha Sudeep, Muhammad Zaheer Saad Ullah, Ahmed Elkady

**Affiliations:** 1 Department of Neurosciences, Tawam Hospital, Al Ain, ARE; 2 Department of Medicine, United Arab Emirates University, Al Ain, ARE

**Keywords:** lambert-eaton myasthenic syndrome, myasthenia gravis (mg), neuromuscular junction disorders, repetitive nerve stimulation, voltage-gated calcium channel antibody

## Abstract

Lambert-Eaton myasthenic syndrome (LEMS) is a rare presynaptic neuromuscular junction disorder that may be mistaken for peripheral neuropathy, radiculopathy, inflammatory myopathy, or myasthenia gravis. We report a female in her early 40s with two months of progressive, predominantly proximal lower-limb weakness, areflexia, and dry mouth, without ocular or bulbar symptoms. Routine laboratory testing and creatine kinase were unremarkable, while spinal imaging showed degenerative abnormalities that did not explain the clinical pattern. Nerve conduction studies demonstrated low compound muscle action potential amplitudes with preserved sensory responses and no demyelinating features. Low-frequency repetitive nerve stimulation produced a decrement, whereas brief exercise caused a marked increase in compound muscle action potential amplitude. Serum P/Q-type voltage-gated calcium channel antibodies were positive, confirming LEMS. Pyridostigmine produced partial symptomatic improvement. Amifampridine was subsequently initiated, followed by intravenous immunoglobulin, after which the patient demonstrated marked improvement in lower-limb strength and walking ability. Initial malignancy screening was negative, and longitudinal surveillance was planned. This case emphasizes that a decrement on low-frequency stimulation is not specific for myasthenia gravis and that low resting motor amplitudes with marked post-exercise facilitation strongly indicate a presynaptic neuromuscular junction disorder.

## Introduction

Lambert-Eaton myasthenic syndrome (LEMS) is a rare autoimmune presynaptic neuromuscular junction disorder caused most often by antibodies against P/Q-type voltage-gated calcium channels. Impaired calcium entry reduces acetylcholine release and produces the characteristic combination of proximal weakness, reduced or absent tendon reflexes, and autonomic symptoms [1].

Diagnosis can be delayed because proximal weakness and low resting compound muscle action potential (CMAP) amplitudes may mimic neuropathy or myopathy. A low-frequency repetitive nerve stimulation (RNS) decrement may also be misinterpreted as evidence of myasthenia gravis (MG). The diagnostic distinction depends on integrating the clinical phenotype with low baseline CMAP amplitudes, post-exercise or high-rate facilitation, and serological findings [2-4]. We describe a patient whose presentation initially suggested inflammatory neuropathy but whose electrophysiological pattern established a neuromuscular junction disorder.

## Case presentation

A female in her early 40s presented to the neurology clinic with a two-month history of progressive heaviness and weakness in both legs. She reported increasing difficulty climbing stairs and rising from a chair and had recently begun using a wheelchair for longer distances. Upper limbs were not involved. She denied pain, sensory symptoms, diplopia, ptosis, dysarthria, dysphagia, dyspnea, sphincter disturbance, or diurnal fluctuation. Dry mouth was the only autonomic symptom. Her medical history was notable for long-standing, poorly controlled diabetes mellitus.

On examination, extraocular movements were full, and neither spontaneous nor fatigable ptosis or diplopia was present. Upper-limb strength was normal. Lower-limb power was reduced predominantly proximally, with Medical Research Council grades of 3/5 to 4/5, while distal strength was preserved. Vibration sensation was reduced at the feet, but pinprick and joint-position sensation were intact. Lower-limb tendon reflexes were absent, upper-limb reflexes were preserved, and plantar responses were flexor.

Complete blood count, renal and liver function tests, thyroid studies, vitamin B12, folate, vitamin D, serum protein electrophoresis, immunofixation, and serum free light-chain ratio were unrevealing. Creatine kinase was normal. Brain magnetic resonance imaging showed nonspecific white-matter changes. Spinal magnetic resonance imaging demonstrated degenerative cervical and lumbar changes, including a right L2-L3 disc extrusion, but these findings did not account for the symmetric proximal weakness.

Neurophysiological findings

Sensory nerve action potentials were preserved. Motor studies showed low CMAP amplitudes in the upper and lower limbs, with normal conduction velocities, distal motor latencies, and F-wave responses (Tables 1, 2). Needle electromyography showed no fibrillation potentials or positive sharp waves in sampled muscles. Motor unit action potential (MUAP) morphology and recruitment were normal, apart from reduced interference in both quadriceps muscles attributable to limited voluntary activation. The absence of demyelinating features, active denervation, or a myopathic motor-unit pattern made chronic inflammatory demyelinating polyradiculoneuropathy, active axonal neuropathy, radiculopathy, and inflammatory myopathy less likely.

**Table 1:** Summary of motor nerve conduction results. Abbreviations: R, right; L, left; B., below; A., above; EDB, extensor digitorum brevis; AH, abductor hallucis; Tib Ant, tibialis anterior; APB, abductor pollicis brevis; ADM, abductor digiti minimi; NR, no response.

Nerve/Sites	Muscle	Latency (ms)	Amplitude (mV)	Dur. (ms)	Segments	Distance (mm)	Lat Diff (ms)	Velocity (m/s)	Dur. 1-5 (ms)	Temp. (°C)
R Peroneal - EDB										
Ankle	EDB	4.5	0.6	6.6	Ankle - EDB				10.73	30.6
B. Fib Head	EDB	11.67	0.6	6.8	B. Fib Head - Ankle	330	7.17	46	11.02	30.6
A. Fib Head	EDB	13.4	0.6	6.4	A. Fib Head - B. Fib Head	80	1.73	46	11.23	30.6
R Tibial - AH										
Ankle	AH	2.73	1.7	6.1	Ankle - AH				11.85	30.6
R Peroneal - Tib Ant										
Fib Head	Tib Ant	3.96	2.1	10.3	Fib Head - Tib Ant				19.33	30.2
Pop fossa	Tib Ant	5.08	1.8	10.4	Pop fossa - Fib Head	60	1.13	53	19.79	30.2
L Peroneal - EDB										
Ankle	EDB	NR	NR	NR	Ankle - EDB				NR	30.6
B. Fib Head	EDB	NR	NR	NR	B. Fib Head - Ankle		NR		NR	30.6
A. Fib Head	EDB	NR	NR	NR	A. Fib Head - B. Fib Head		NR		NR	30.6
L Tibial - AH										
Ankle	AH	4.06	1.2	5.3	Ankle - AH				8.96	33.7
L Peroneal - Tib Ant										
Fib Head	Tib Ant	3.25	2.1	14	Fib Head - Tib Ant				21.81	30.6
Pop fossa	Tib Ant	4.38	2	11.4	Pop fossa - Fib Head	60	1.13	53	22.08	30.6
R Median - APB										
Wrist	APB	4.98	3.4	7.6	Wrist - APB				17.17	32.3
Elbow	APB	9.73	3.4	8.3	Elbow - Wrist	235	4.75	49	16.29	32.5
R Ulnar - ADM										
Wrist	ADM	2.42	5.4	6	Wrist - ADM				14.19	33.7
B. Elbow	ADM	7.04	5.1	6	B. Elbow - Wrist	240	4.63	52	13.81	33.7
A. Elbow	ADM	8.46	5.1	6.4	A. Elbow - B. Elbow	75	1.42	53	14.19	33.6
L Median - APB										
Wrist	APB	4.5	3.1	6.7	Wrist - APB				24.83	32.9
Elbow	APB	9.17	2.6	6.2	Elbow - Wrist	235	4.67	50	14.04	31.1
L Ulnar - ADM										
Wrist	ADM	2.73	5.6	6.7	Wrist - ADM				23.81	31
B. Elbow	ADM	7.19	5.1	7.6	B. Elbow - Wrist	240	4.46	54	14.92	32.8
A. Elbow	ADM	8.67	5.2	7.9	A. Elbow - B. Elbow	80	1.48	54	25.17	32.8

**Table 2:** Summary of sensory nerve conduction study findings. Abbreviations: SPN, superficial peroneal nerve; NP Amp, negative peak amplitude.

Nerve/Sites	Rec. Site	Onset Lat (ms)	Peak Lat (ms)	NP Amp (µV)	Segments	Distance (mm)	Velocity (m/s)	Temp. (°C)
Lower limbs - Right								
SPN Lat leg	Ankle	2.45	3.23	5.6	SPN Lat leg - Ankle	130	53	31.3
Sural Calf	Lat Ankle	1.88	2.76	22.4	Sural Calf - Lat Ankle	115	61	31
Lower limbs - Left								
SPN Lat leg	Ankle	2.45	3.33	5.6	SPN Lat leg - Ankle	140	57	30.6
Sural Calf	Lat Ankle	2.81	3.8	25.2	Sural Calf - Lat Ankle	140	50	30.6
Upper limbs - Right								
Median Wrist	Digit II	3.59	4.74	26.9	Median Wrist - Digit II	130	36	32.3
Median Wrist	Digit III	3.59	4.79	27.8	Median Wrist - Digit III	130	36	32.3
Median Palm (Mixed N)	Median Wrist	2.5	3.28	33	Median Palm (Mixed N) - Median Wrist	80	32	32.7
Ulnar Wrist	Digit V	2.19	3.13	30.2	Ulnar Wrist - Digit V	110	50	32.6
Ulnar Palm (Mixed N)	Ulnar Wrist	1.61	2.14	19.7	Ulnar Palm( Mixed N) - Ulnar Wrist	80	50	32.9
Radial Forearm	Wrist	1.88	2.6	36.3	Radial Forearm - Wrist	100	53	33.9
Upper limbs - Left								
Median Wrist	Digit II	3.54	4.58	27.8	Median Wrist - Digit II	130	37	30.8
Median Wrist	Digit III	3.8	4.84	23.2	Median Wrist - Digit III	130	34	31.2
Median Palm (Mixed N)	Median Wrist	2.5	3.18	29.1	Median Palm (Mixed N) - Median Wrist	80	32	31.3
Ulnar Wrist	Digit V	1.98	2.76	25.7	Ulnar Wrist - Digit V	110	56	31.6
Ulnar Palm (Mixed N)	Ulnar wrist	1.46	1.98	21.6	Ulnar Palm (Mixed N) - Ulnar Wrist	80	55	31.7
Radial Forearm	Wrist	1.72	2.4	39.5	Radial Forearm - Wrist	100	58	31.6

**Figure 1:**
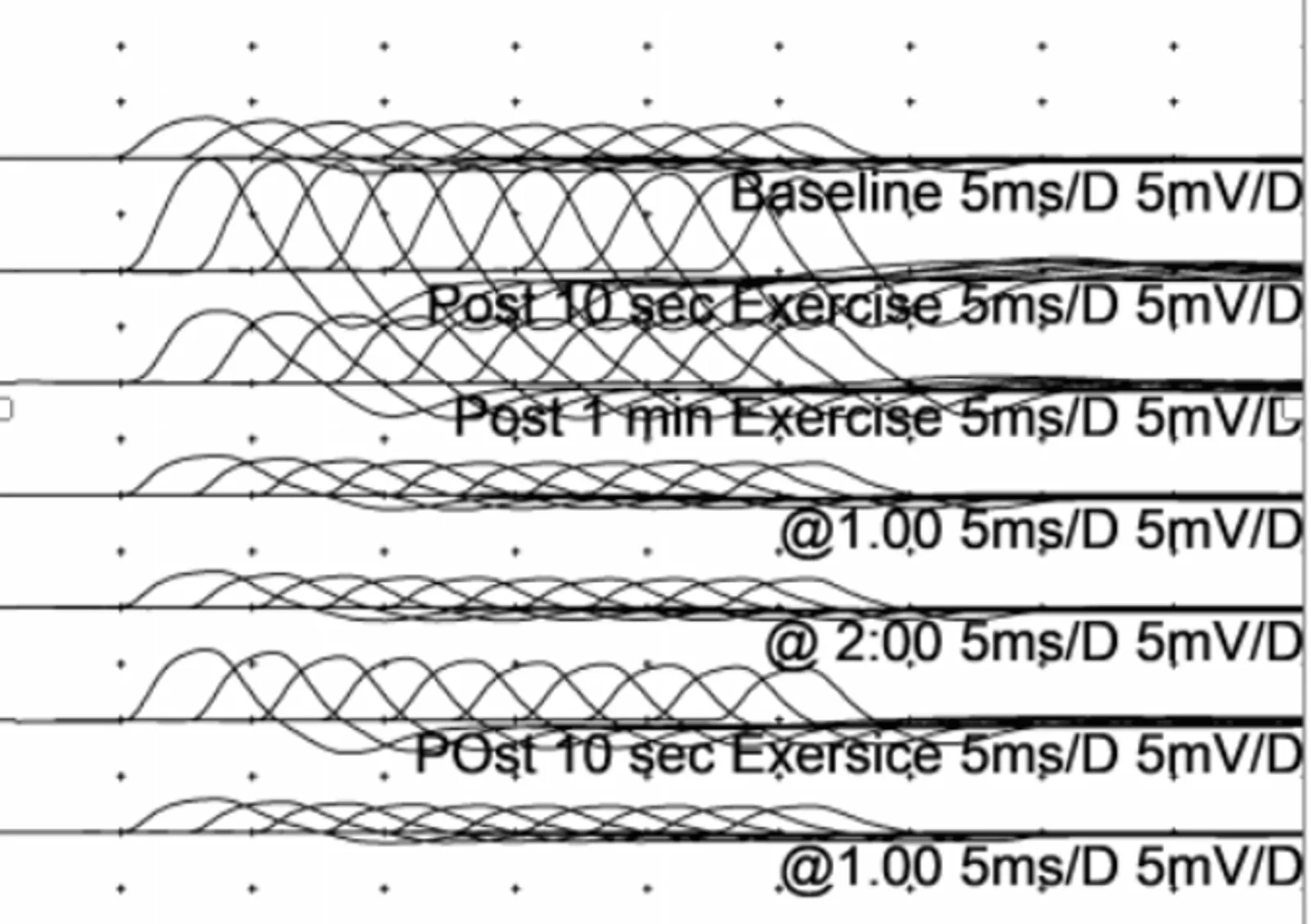
Low-frequency repetitive nerve stimulation of the median nerve showing significant decrement at baseline, immediate post-exercise facilitation, and subsequent post-exercise exhaustion.

Low-frequency RNS of the median nerve showed significant decrement, followed by immediate post-exercise facilitation and subsequent post-exercise exhaustion, prompting the suggestion of MG (Table 3 and Figure 1).

**Table 3:** Repetitive nerve stimulation (RNS) from right median abductor pollicis brevis.

Train	Rate (Hz)	Amplitude (mV)	▲ Amp 4-1 (%)	Fac Ampl (%)	Area (mVms)	▲Area 4-1 (%)	Fac Area (%)
Baseline	3	3.3	18.7	100.0	14.1	22.2	100.0
Post 10-sec exercise	3	6.2	5.3	187.1	23.6	6.0	167.9
Post exercise @ 1.00 min	3	5.6	3.1	169.3	23.4	4.7	166.3
Post exercise @ 2:00 min	3	3.4	15.2	101.9	15.6	16.9	111.1
Post exercise @ 3:00 min	3	3.0	13.9	92.0	12.9	14.9	91.6
Post exercise @ 5:00 min	3	3.1	18.3	93.5	13.5	23.2	96.3

**Figure 2:**
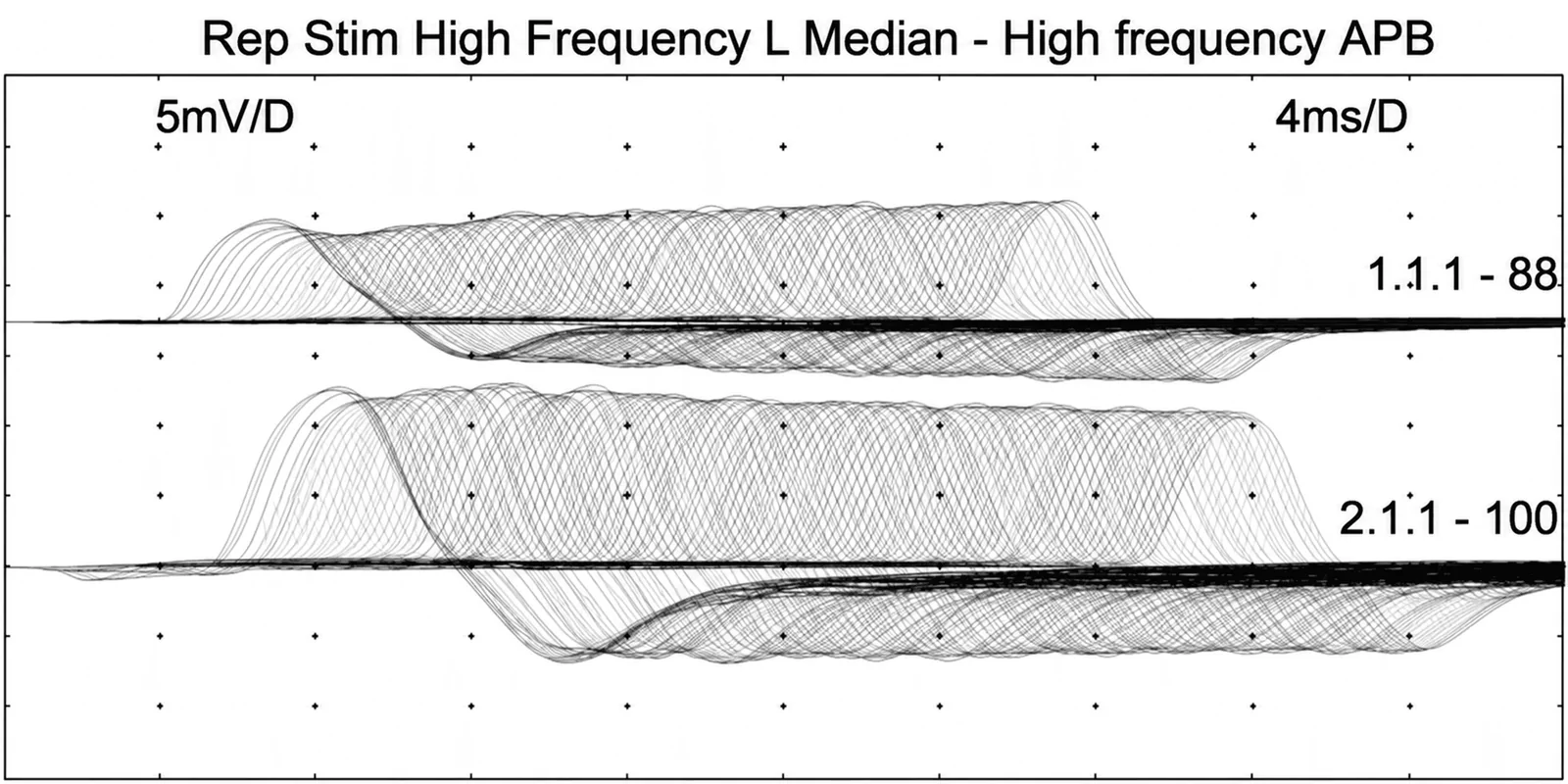
Repetitive nerve stimulation at 10 Hz showing an incremental compound muscle action potential response, supporting a presynaptic neuromuscular junction disorder. APB: abductor pollicis brevis; L, left; Rep Stim: repetitive nerve stimulation.

However, as the patient's clinical findings did not fit with typical MG, she was recalled for further testing. Repeat median motor testing demonstrated a baseline CMAP amplitude of 3.6 mV in the abductor pollicis brevis. After 10 seconds of exercise, the amplitude increased to 10.0 mV, representing an approximately 178% increment, or a 2.8-fold increase from baseline. Facilitation persisted at 30 seconds before the response moved toward baseline. Low-frequency stimulation at 3 Hz produced an approximately 14% decrement. The patient could not tolerate 50-Hz stimulation; however, 10-Hz stimulation also demonstrated an incremental response (Table 4 and Figure 2).

**Table 4:** Results of repetitive nerve stimulation (low- and high-frequency stimulation).

Anatomy/Train	Rate (Hz)	Amplitude (mV)	▲ Amp 4-1 (%)	Fac Ampl (%)	Area (mVms)	▲Area 4-1 (%)	Fac Area (%)
Baseline	3	3.6	14.2	100.0	14.5	16.1	100.0
Post 10-sec exercise	3	10.0	5.0	279.7	35.0	7.6	240.8
Post 1-min exercise	3	6.3	6.4	176.5	27.6	14.1	190.0
@ 1.00	3	3.5	13.4	97.4	16.0	16.1	110.1
@ 2:00	3	3.2	13.9	88.5	14.0	20.4	96.3
Post 10-sec exercise	3	6.4	14.1	179.1	21.8	11.8	149.7
@ 1.00	3	3.0	15.8	83.0	13.4	18.9	92.0
Baseline	10	3.0	10.1	-12.3	13.0	18.6	13.6
Post ex 10 seconds	10	5.1	2.9	15.6	18.9	22.8	31.4
@ 1:00	2	2.9	17.6	17.9	13.1	15.2	16.8
@ 1:00	10	2.9	16.6	21.5	13.5	33.9	39.1

Serology, diagnosis, treatment, and follow-up

Subsequently, her serum P/Q-type voltage-gated calcium channel antibodies came back positive. Acetylcholine receptor antibodies and muscle-specific kinase antibodies were negative. SOX1 antibodies and the remainder of the paraneoplastic antibody panel were also negative. The clinical phenotype, low resting CMAP amplitudes, marked post-exercise facilitation, and antibody positivity established the diagnosis of LEMS.

Pyridostigmine produced partial symptomatic improvement. Amifampridine was subsequently initiated, followed by intravenous immunoglobulin (IVIG), after which she demonstrated marked improvement in lower-limb power and walking ability. The patient was a smoker. Positron emission tomography-computed tomography and oncology evaluation did not identify an underlying malignancy. Despite the negative initial malignancy evaluation and negative SOX1 and paraneoplastic antibodies, continued malignancy surveillance was planned because of her smoking history, the recognized association between LEMS and small-cell lung cancer, and the fact that LEMS can precede the detection of small-cell lung cancer.

## Discussion

This case highlights an important diagnostic pitfall: electrophysiological findings, including repetitive nerve stimulation, should not be interpreted as isolated positive or negative results. Instead, accurate diagnosis requires integration of the clinical phenotype with the complete electrophysiological pattern to distinguish disorders of neuromuscular transmission from neuropathic or myopathic processes.

For starters, Low CMAP amplitudes can also be misinterpreted as diffuse axonal neuropathy. However, axonal neuropathies are typically accompanied by neurogenic abnormalities on needle electromyography, including fibrillation potentials, positive sharp waves, and chronic motor-unit remodeling [5]. Their absence, particularly in the presence of preserved sensory responses and normal conduction velocities, should prompt reconsideration of the diagnosis and further assessment for a neuromuscular junction disorder [5,6]. In LEMS, the CMAP amplitude is low because insufficient acetylcholine is released at rest, not because motor axons have necessarily been lost. Demonstrating a substantial increment after 10 seconds of exercise is therefore diagnostically valuable. Ten-second exercise has been reported to be more sensitive than longer exercise for post-exercise facilitation [4]. Reflex facilitation after brief voluntary contraction may provide an additional bedside clue, although it is not consistently present [7].

Importantly, a decrement during low-frequency RNS is not specific for MG. Low-frequency stimulation may produce a significant decrement in both postsynaptic MG and presynaptic LEMS [1-4]. Therefore, the presence of decrement alone establishes impaired neuromuscular transmission but does not reliably localize the defect to the pre- or postsynaptic membrane. In postsynaptic disorders like MG, resting CMAP amplitudes are usually normal or near normal, and post-exercise repair is generally modest. In contrast, presynaptic LEMS is characterized by reduced resting CMAP amplitudes and marked facilitation following brief exercise or high-frequency stimulation, reflecting calcium accumulation within the presynaptic nerve terminal and increased acetylcholine release [1-4]. Thus, interpretation of low-frequency RNS must be integrated with baseline CMAP amplitude, post-exercise facilitation, the clinical phenotype, and serological findings. The electrophysiological distinctions between MG and LEMS are summarized in Table 5.

**Table 5:** Key electrophysiological distinctions between myasthenia gravis and Lambert-Eaton myasthenic syndrome. CMAP: compound muscle action potential; EMG: electromyography; RNS: repetitive nerve stimulation.

Feature	Myasthenia gravis	Lambert-Eaton myasthenic syndrome
Baseline CMAP amplitude	Usually normal or near normal	Often reduced
Low-frequency RNS (2-5 Hz)	Typical decrement	Decrement may occur
Pattern during the train	Often early, nonprogressive, or U-shaped	May progressively worsen
Immediately after exercise	Transient partial repair may occur	Marked facilitation is characteristic
High-frequency RNS	No substantial increment in most cases	Marked increment/facilitation
Single-fiber EMG	Increased jitter	Increased jitter

The patient had several features that favored LEMS: leg-predominant proximal weakness, lower-limb areflexia, dry mouth, low resting CMAP amplitudes, a large post-exercise increment, and P/Q-type voltage-gated calcium channel antibodies. The absence of ocular and bulbar symptoms also supported LEMS, although ocular manifestations can occur and their absence is not diagnostic. Subtle ocular signs, including paradoxical lid elevation with sustained upgaze, have been reported in LEMS [8].

Diabetes and degenerative spinal imaging were important diagnostic distractors. Although diabetic distal symmetric polyneuropathy could account for the reduced distal vibration sense and absent ankle reflexes, it does not explain the rapid progression of symmetric proximal weakness or the characteristic post-exercise facilitation. Diabetic lumbosacral radiculoplexus neuropathy was also considered because of the patient's diabetes and proximal leg weakness. However, it typically presents with severe unilateral pain involving the hip, thigh, or buttock, followed by asymmetric weakness, muscle wasting, weight loss, and areflexia, with needle electromyography demonstrating active denervation and other neurogenic changes, none of which were present in this patient [9,10].

Similarly, the L2-L3 disc extrusion was considered an incidental finding rather than the cause of the patient's symptoms. Clinically significant upper lumbar radiculopathy would be expected to produce pain and neurological deficits in a corresponding root distribution, often with asymmetric weakness, sensory disturbance, or reflex changes. Electrophysiological confirmation typically requires needle electromyographic evidence of a myotomal process, including fibrillation potentials, positive sharp waves, or chronic neurogenic motor-unit changes, which were absent in this case. This highlights the importance of correlating imaging findings with the clinical and electrophysiological pattern rather than assuming structural abnormalities are responsible for the presenting syndrome [11].

Management has three parallel goals in LEMS. First, amifampridine is the principal symptomatic treatment because potassium-channel blockade prolongs presynaptic depolarization, increases calcium entry, and enhances acetylcholine release [12]. Pyridostigmine may provide additional but usually limited benefit. Second, patients with functionally significant autoimmune disease may require immunotherapy, including intravenous immunoglobulin and, in selected cases, longer-term immunosuppression; plasma exchange may be considered for short-term rescue [13,14]. Third, all patients require careful assessment for an underlying malignancy, particularly small-cell lung cancer [1,14,15]. Risk assessment should incorporate age, smoking exposure, clinical phenotype, SOX1 antibody status, and validated prediction tools such as the Dutch-English LEMS Tumor Association Prediction (DELTA-P) score [14,15]. In our patient, smoking exposure increased concern for an occult malignancy, although initial PET-CT and oncology assessment were negative and SOX1 and the remainder of the paraneoplastic antibody panel were negative. Continued malignancy surveillance was therefore planned despite the negative initial evaluation.

The main limitation of this report is the lack of long-term follow-up at the time of writing. Ongoing observation is needed to document the durability of neurological improvement, access and response to amifampridine, and the results of serial malignancy surveillance.

## Conclusions

LEMS should be considered in patients with subacute proximal leg-predominant weakness, reduced or absent reflexes, autonomic symptoms, and low resting CMAP amplitudes. A low-frequency RNS decrement is not specific for MG. Marked post-exercise or high-rate facilitation, particularly when combined with P/Q-type voltage-gated calcium channel antibodies, identifies a presynaptic neuromuscular junction disorder. Careful clinicophysiological correlation can prevent misdiagnosis as neuropathy, radiculopathy, or myopathy and enables timely symptomatic treatment, immunotherapy, and malignancy surveillance.
